# Molecular Mechanisms of Phthalate-Induced Hepatic Injury and Amelioration by Plant-Based Principles

**DOI:** 10.3390/toxics13010032

**Published:** 2025-01-02

**Authors:** Lalit Kumar Singh, Rashmi Pandey, Nikhat Jamal Siddiqi, Bechan Sharma

**Affiliations:** 1 Department of Biochemistry, University of Allahabad, Prayagraj 211002, Uttar Pradesh, India; mylalitkumar@gmail.com; 2 Department of Biochemistry, Government Medical College, Haridwar 247667, Uttarakhand, India; 3 Department of Internal Surgical Nursing, College of Nursing, King Saud University, Riyadh 11421, Saudi Arabia

**Keywords:** phthalates, hepatotoxic, oxidative stress, metabolic disorder, polyphenols

## Abstract

Phthalates are the emerging environmental toxicants derived from phthalic acid and its constituents, which are moderately present in plastics and many personal care products. Phthalate exposure occurs through various environmental factors, including air, water, and soil, with absorption facilitated via ingestion, inhalation, and dermal contact. Upon exposure, phthalates become bioavailable within the biological systems and undergo biotransformation and detoxification processes in the liver. The physicochemical properties of phthalates indicate their lipophilicity, environmental persistence, and bioaccumulation potential, influencing their absorption, distribution, and hepatic biotransformation. The prolonged exposure to phthalates adversely influences the biological redox system by altering the levels of the enzymatic and non-enzymatic antioxidants, molecular signaling pathways, and causing hepatic pathogenesis. The strategies to combat phthalate-induced toxicity include avoiding exposure to these compounds and using plant-based bioactive molecules such as polyphenols, which possess therapeutic potential as antioxidants, suppress inflammatory cascades, prevent oxidative damage, and stabilize cellular integrity. This review presents a comprehensive and updated account of the chemical, biochemical, immunological, and toxicological properties of phthalates, along with novel plant-based therapeutic strategies to mitigate the phthalate-induced adverse effects on living systems.

## 1. Introduction

Plastics and their constituents have greatly benefited society since it was invented in 1907. However, they have many negative impacts on the environment and health of animals and humans, which has raised a serious global concern. People are constantly exposed to plastics via contaminated food, packaging leachate (e.g., water bottles and medical devices), atmospheric fallout and urban dust containing microplastics, personal care products (PCPs) (e.g., cosmetic packaging), and synthetic clothing [1,2]. According to some workers, about 3 million tons of phthalic esters per year are produced worldwide [3,4]. It may exceed up to 500 million metric tons by 2050. Most of them will be single-use phthalate-mediated products. These esters can be found in everyday products, such as PVCs, plastic bags, perfumes, food packaging, lubricants, cosmetics, toys, and industrial paints, as well as blood transfusion packs [5,6].

According to Josh et al. (2012) [7], the degree of flexibility or plasticity in plastics increases with the concentration or percentage of phthalate rise. The most widely used phthalate is DEHP. Phthalates are classified as endocrine-disrupting chemicals (EDCs); dibutyl phthalate (DBP), mono-ethyl hexyl phthalate (MEHP), diisononyl phthalate (DiNP), diethyl phthalate (DEP), DEHP, and dicyclohexyl phthalate (DCHP) were tethered in 1920.

Phthalates are not a family of natural compounds; they are man-made or synthetic xenobiotic compounds that have become an integral part of modern human life. In large amounts, they are synthesized in different laboratories and industries for softening plastics. Phthalates are a group of chemicals used for increasing the durability of plastics, electric wire, home appliances, transparency, flexibility, durability, thermal stability, and softening of plastics known as plasticizers (substances used with polyvinyl chlorides (PVCs) for plastics). These phthalates are used in plastics as a plasticizer for food packaging for a long period for packaging medical devices and medicines and for preventing household and electronic instruments from pre-fire [8,9,10].

Based on the molecular weight, phthalates have been divided into two classes: (1) high molecular weight (HMwP) phthalates, such as di-ethyl hexyl phthalates (DEHPs), di-cyclohexyl phthalate (DCHP), di-isodecyl phthalates (DiDPs), and diisononyl phthalate (DiNP), used for the production of plastics containing products such as packaging of medical devices and food, and preventing household and electronic instruments from pre-fire [8,9,10,11], and (2) low molecular weight phthalates (LMwPs), like benzyl butyl phthalate (BBzP) and diethyl phthalate (DEP), used for the assembling of daily use beauty care products (such as shampoo, cosmetics, perfume, and lotions), adhesives, and paints [12].

The exposure paths, industrial usage, and environmental behavior of these two different molecular weight phthalates are different. Phthalates can convert into their respective monoesters within a few hours, can bioaccumulate, and cannot be easily excreted out. Phthalates are non-covalently bound with plastics and consequently can easily leach into the environment through air, soil, water, food, heat, beverages, and drinks. These environmental factors (air, soil, water) [13,14,15] act as a career for phthalate dispersion and induce different types of chronic diseases into the human body. Phthalates enter the human body either through digestion, inhalation, or dermal absorption [16,17,18]. Phthalates’ perseverance in the environment poses an emerging public health risk, potentially impacting reproduction, development, obesity, functioning organs (like the kidney, brain, liver), and other health concerns [2].

Overall, phthalates have aroused concern about their potential adverse effect on both the environment and living organisms. Their ubiquitous use in consumer products has led to their presence in the microenvironment, where they can persist and bioaccumulate. Phthalates can leach out of the environment, even if they can undergo a metabolic system to excrete out of the living body and accumulate in tissue over time. Due to the accumulation, they can be characterized by reproductive toxicity in people and animals, can induce infertility and reproductive issues in males, including decreased sperm quality and reproductive developmental abnormalities, and may adversely affect the liver, kidneys, lungs, and neurological systems. Due to the lipophilic nature of phthalates, they easily accumulate for long periods in the adipose tissue of animals [19] and induce different types of diseases, like non-alcoholic fatty liver disease (NAFLD), obesity, and negative health concerns.

There is a plethora of current studies that provide additional details regarding the interplay of many factors in the development of liver disease. The “two-hit” hypothesis is the most widely accepted theory, in which the first hit suggested that the accumulation of fat in the liver, which induces inflammation, results in cirrhosis and non-alcohol steatohepatitis. However, the effect of the second hit remains unclear; it could be fatty acid oxidation, due to generated free radicals that damage the liver [20], even though the “two-hit” hypothesis is not suitable for liver damage because numerous manifestations generate liver damage by different metabolic pathways, insulin resistivity, environmental exposures, fatty acid synthase pathways, and genetic predispositions [21,22].

In this article, a comprehensive review of different aspects of phthalates, including their entry into the environment via the mechanism of exposure to the living systems, toxicity, and perturbations in the cellular, biochemical, molecular, and signaling systems, are presented. Additionally, the review discusses potential strategies for managing the challenges associated with the widespread application of phthalates and concludes with proposed mitigation strategies involving the application of plant-based principles.

## 2. Chemical Composition of Phthalate

**Figure 1:**
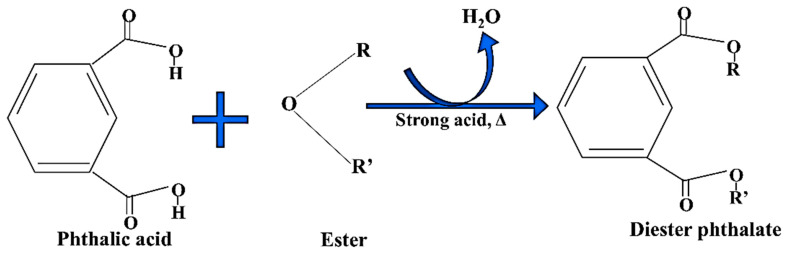
The structure and synthesis of phthalate. Phthalic acid undergoes esterification with alcohol in the presence of an acid catalyst, producing phthalate esters and water as a byproduct.

Phthalates are synthesized through the reaction of alcohols with the carboxyl functional groups attached to the benzene ring of phthalic acid [23,24] (Figure 1).

Phthalates are the esters of benzene-1,2-dicarboxylic acid (C_8_H_4_O_4_^−2^) produced by the addition of a branched or linear chain of alcohol to phthalic anhydride in the presence of a catalyst. Phthalates are the esters of phthalic acid, produced by the deprotonation of both the carboxyl groups of phthalic acid, resulting in a molecular weight of 164.11. The diversity of phthalates is produced by adding an alcohol chain (linear or branched) to the phthalic acid (to the diester groups of the phthalic acids) [25]. Both linear and branched phthalate esters are used in the manufacturing of plastics, with mainly linear esters providing maximum flexibility at low temperatures with low volatility, stability, and fluidity for higher molecular mass phthalate esters, which make them fancier plasticizers. Less than C_6_ at their alkyl side chain is generally not used as a plasticizer because they are more volatile. The alkyl chain of these acid esters forms the non-covalent bond with PVC, which means they are present in free form in the plastics. Due to this, plastics are soft and flexible. Based on the molecular weight, the phthalates are classified into two classes: (1) high molecular weight phthalates (HMwPs), and (2) low molecular weight phthalates (LMwPs).

### 2.1. Based on Molecular Weight Phthalates Are Classified into Two Classes

#### 2.1.1. High Molecular Weight Phthalates (HMwPs)

HMwPs improve the enforcement of plastics, especially in appliances where resilience and persistence are important. Phthalates are formed by the reaction between phallic anhydride and alcohol in the presence of a catalyst. These phthalates have larger molecular structures with longer chains of carbon atoms (C_7_ to C_13_) along with alcohol moieties, such as DCHP (C_20_H_26_O_4_), di-isononyl phthalate [DiNP (C_26_H_42_O_4_)], and DEHP (C_24_H_38_O_4_). The high molecular weight phthalates are synthesized by a chemical process to form larger molecules with the required properties for the customization of plastics. The high boiling (>250 °C) and low melting points (−25 °C to −55 °C) of phthalates make them appropriate for applications such as heat-transfer carriers and fluids. These physiochemical properties depend on their carbon length and chemical structure [26]. Phthalates are colorless as well as odorless at 25 °C. In general, compounds with longer carbon chains tend to be more hydrophobic and have higher partition coefficients (Log P, K_OW_) in nonpolar phases, like octanol or air, compared to water. This trend is frequently observed due to reduced polarity and greater van der Waals interactions in comparison to shorter-chain molecules. According to Bauer and Herrmann (1998) [27], LMwPs exhibit weaker hydrophobicity compared to HMwPs, as indicated by their partition coefficients (LogK_OW_) (Table 1). Additionally, Asakura et al. (2004) [28] observed that LMwPs are more radially leached from waste than the more hydrophobic HMwPs. These partition coefficients are important in finding the behavior and environmental fate of PAEs [29], including their potential for long-term transportation and bioaccumulation, and in informing agencies regarding the health risk assessments and administrative stratagems for these phthalates in the environment [30].

#### 2.1.2. Low Molecular Weight Phthalates (LMwPs)

LMwPs have shorter carbon chain lengths (three to six carbon chains), which leads to lower molecular weights. LMwPs are generally more volatile and offer transient plasticization. These are recurrently utilized where long-term solidity is less vital or requires brief flexibility. LMwPs are of greater concern for health issues in an organism as compared to HMwPs; this is because these low molecular weight phthalates tend to leach out into the environment and lead to a higher exposure level. Due to these concerns, regulatory authorities restrict the use of these phthalates as compared to HMwPs.

Because of their propensity to cause hepato-toxicity, reproductive damage, and endocrine disruption, LMwPs have been a subject theme for intensive research. Due to these concerns, some LMwPs have been proscribed or regulated in some daily-use consumer products. HMwPs have a lower bioavailability and are considered less hazardous to the environment and health than the LMwPs. However, they are tranquil, subject to regulatory oversight, and maybe a concern in specific circumstances [31]. Thus, both the HMwPs and LMwPs are used as plasticizers in a variety of products; their difference in physical properties, molecular structure, and characteristics creates discrepancies in applications, regulatory status, and environmental consequences, and can have possible adverse effects on liver health such as liver toxicity [32], metabolic disorders such as dyslipidemia, insulin resistance, obesity and cardiovascular diseases, inflammation, and oxidative stress, resulting in the development of more severe conditions such as fatty liver disease and liver tumorigenesis.

## 3. Sources of Phthalates Exposure

### 3.1. Exposure of Phthalate Through Water

The leaching of phthalates can occur during the production, use, and disposal of plastic items and the breakdown of plastics in ecological environments, such as rivers, oceans, and soil. During the rainy season, due to changes in physiological conditions such as pH, and temperature, these phthalates leach out and mix with groundwater so that most of the population is directly exposed to the phthalic acid esters (PAEs) in the ground-level drinking water. These phthalic acids ingested through water cause structural changes in the receptors of the endocrine system and tempt the antagonistic expression of these receptors. Consequently, they induce metabolic perturbations, poor development of organs, and neurological ataxia in the organisms [21]. Since phthalates are usually found in various consumer products, including water bottle containers, prolonged storage or exposure to heat may cause them to leach out of the bottle containers and propagate into the organism. When organisms ingest different beverages or water stored in phthalates-containing containers, they have the potential for these chemicals to enter the body. These migrating water bottles are synthesized with various monomers of phthalates, and these phthalates migrate from the bottle to the organism and affect various organs [33]. The drinking water threshold level values for DEP, DBP, DMP, and DEHP have been established by the United States Environmental Protection Agency (USEPA) at 0.55, 0.45, 5.0, and 5.0 mg/L (mg/L is also known as part per million, ppm), respectively. The WHO (2008) guidelines state that water should have a phthalate concentration of 6–8 µg/L. Due to the hydrophobic nature of phthalates, they can cross biological barriers, including the placenta of the mother, which can directly induce adverse effects on neonates and introduce toxicity [34,35].

### 3.2. Exposure of Phthalate Through Air

According to some workers [36,37], phthalates are disseminated around the planet due to atmospheric transportation over large distances, whereas phthalate esters are distributed globally in different amounts from remote sense areas to the Amazon rainforests to the Arctic. Phthalate exposure through air can occur via various sources, including indoor and outdoor environments, as well as through the inhalation of airborne particles and dust. Indoor air concentrations of phthalates are tenfold higher than outdoor levels [38]. The concentration of indoor phthalate is temperature dependent; as the temperature rises the phthalates leach out into the surroundings but diminish as the humidity increases. In the cold environment, the phthalates’ concentration reaches up to 0.05 mg/m^3^. However, long-term exposure and a rise in temperature can show detrimental impacts on the organisms. The WHO’s 2003 guidelines display the range of phthalate concentration in urban air from 5 to 132 ng/m^3^. LMwPs are used in sealing, wall paint, building materials, and daily use products, such as nail polish, perfume, and home appliances. However, HMwPs are used in wall coverings, cleaning agents, and floor or plastic furniture. So, these phthalates are present in higher concentrations in household pollution [31,39,40]. Human health is significantly impacted by indoor air and dust because they spend most of their time inside the houses, where phthalate exposure is higher. Newborns and youngsters may be more susceptible to respiratory problems due to their high inspiration rate [41,42,43]. Developing nations utilize more phthalate-containing products because of the accessibility of these packages (toys, containers, and packaging) at low cost. These plastic-based products contribute to indoor exposure to phthalic esters. Developing countries have fewer stringent rules or enforcement measures in place to minimize the use of phthalates in consumer products and other building materials than developing countries, leading to higher levels of phthalate-containing items in the indoor environment [44].

### 3.3. Exposure of Phthalate Through Soil

The concentration of phthalic acid esters in urban areas is higher than in rural areas due to the extensive use of plastics and industrial activities. However, sludge and plastic films are the primary sources of phthalates in rural areas [45]. Phthalates bind non-covalently with the plastics. They physically interact with the chemical ingredients of plastics, which allow them to be easily released into the environment through leaching, migration, and evaporation. The exposure and concentration of phthalates are less in residential, non-industrial, non-cultivated, and roadside areas than in the electric/plastic manufacturing industries. The contents of phthalates like BBP, DMP, and DEP vary in different soils of the earth [46]. Phthalates have leaching properties, so they leach into the soil. The bioaccumulation of different phthalate esters magnifies into crops, plants, humans, and many other organisms. It has been shown that the human population suffers from numerous health issues due to hepatic, gastrointestinal, neurological, and metabolic disorders [45,47].

Some studies have demonstrated that air exposure can show a significant level of phthalate metabolites in urine, indicating absorption and bioaccumulation in the organisms. Air exposure has been associated with various respiratory diseases, developmental toxicity, and endocrine disruption, making it the most concerning route for health [48,49]. Pregnant women, children, and people living in poorly ventilated areas are particularly vulnerable to air-mediated contamination [49].

Phthalate exposure through the air is the most significant route for phthalate toxicity, particularly in indoor environments, followed by waterborne exposure in areas with contaminated water sources. While soil contamination is still a concern in specific environments, it poses a lower risk to overall public health [50].

**Figure 2:**
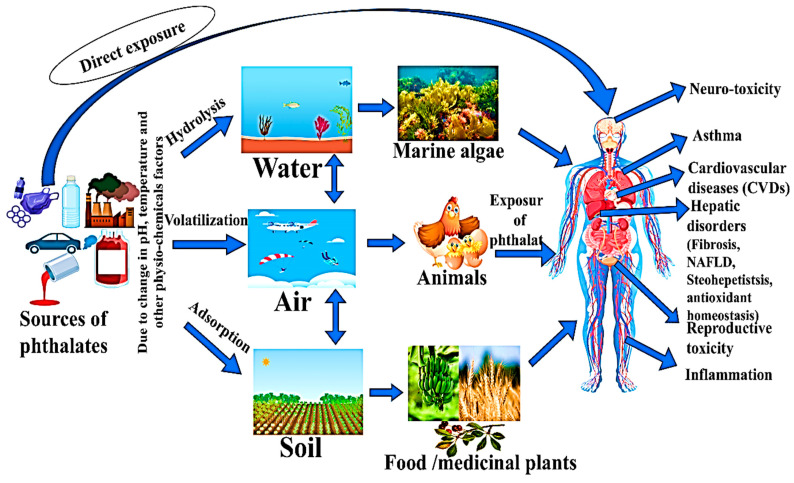
Pathways and routes of human exposure to phthalates released from different sources in the environment.

LMwPs can easily be ingested as compared to HMwPs because LMwPs can easily leave the surface from their sources. HMwPs are primarily introduced into the body through food intake. In contrast, LMwP metabolites are more likely to result from exposure to non-dietary sources [51]. The dermal absorption of phthalates from hand wipes is lower than the total uptake via air, dust, and personal care products [31]. The exposure routes of different phthalates are presented in Figure 2.

## 4. Toxicity and Levels of Phthalates in Biological Matrices

Phthalates exhibit low acute toxicity in rodent studies with LD_50_ values ranging from 1 to 30 g/kg body weight, predominantly affecting the reproductive, endocrine, urinary, and hepatic systems [52]. They are significantly associated with reproductive and developmental toxicity in both animals and humans. For example, maternal exposure to DBP at 100mg/kg body weight/day in rodent models may impair fetal development [53]. The NOAEL (no-observed-adverse-effect level) for DEHP in humans is established at 4.8 mg/kg body weight/day, while the TDI (tolerable daily intake) is set at 48 g/kg of body weight/day. LMwPs (like DEP) can induce severe problems in the skin, mucus of oral and nasal cavities, and conjunctiva [53,54]. HMwPs have been shown to influence the methylation of impaired genes, spermatogenesis, and estrogen responses. Studies in male rodents indicate that low-dose DEHP exposure during puberty significantly affects the function of the nervous system [55,56]. In humans, phthalate exposure has been linked to changes in gene and phenotypic expressions. The epidemiological evidence associated with metabolic disorders includes reproductive toxicity [57]. The cumulative risk assessment, which aggregates hazard quotients (HQs) into a hazard index (HI), is essential for evaluating phthalate exposure. Researchers highlighted that most studies on phthalate (DEHP) and DBP account for a significant proportion of HI, emphasizing the importance of comprehensive risk evaluations [2].

Phthalates are a class of ubiquitous chemicals, primarily known as plasticizers, and are widely incorporated into consumer products, resulting in extensive human exposure. This pervasive use has led to the identification of phthalate metabolites in a broad spectrum of biological matrices, including urine, blood, and serum. Biomonitoring studies have consistently detected metabolites, such as mono-2-ethylhexyl phthalate (MEHP), monoisononyl phthalate (MiNP), mono-n-butyl phthalate (MBP), and mono-ethyl phthalates (MEPs), across diverse populations [57]. The concentration of these metabolites in human samples exhibits variability, influenced by factors like population demographics, geographical location, and environmental sources of exposure. These phthalate metabolite levels are reflective of the magnitude and routes of exposure, including both environmental and dietary sources.

The biomonitoring of phthalate metabolites at different concentrations is an important marker for assessing exposure with implications for public health. Notably, phthalates have been implicated in endocrine disruption and adverse developmental effects, underscoring the need for diligent monitoring. The data presented in Table 2 indicate the presence of phthalate metabolites in biological samples and highlight the widespread contamination in human populations. Such monitoring is crucial for elucidating the exposure trends, informing regulatory policies, and mitigating the public health burden associated with phthalate exposure.

## 5. Metabolism of Phthalates in Liver

**Figure 3:**
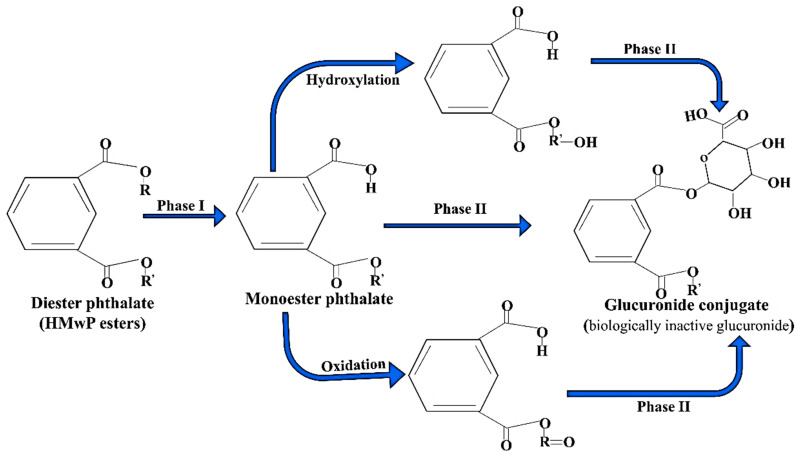
Systematic presentation of metabolic pathways of phthalates in the liver, focusing on the conversion of diester phthalates (HMwP esters) into the corresponding monoester phthalates (LMwP esters). The hydroxylation and oxidation reactions of diesters are catalyzed by the phase I enzymes, while in this context the conjugation reactions of phase II are catalyzed by UDP-glucuronyl transferase.

In the organism, various types of enzymes such as esterase and lipase are present, which are responsible for the hydrolysis of complex forms of phthalates into their monoesters [78]. This monomeric form of phthalate ester is more active than its parent ester. The monomeric form of phthalate can be easily oxidized or hydrolyzed by cyt. P_450_ and other phase I enzymes after absorption into the cell. When these oxidized or simple monomeric forms of phthalate reach into the body, they undergo metabolization. The glucuronic moiety attached to the phthalate by the enzyme uridine 5′-diphospho-glucuronosyltransferase (UGT) forms a biologically inactive glucuronide conjugate and eliminates these toxicants from the body [43,78,79,80,81,82,83,84]. The short-chain phthalate gets easily hydrolyzed into its monoester and hence can be easily excreted through the urine, while long-chain (HMwPs) phthalates pass through many additional biological processes, such as oxidation and hydroxylation, to eliminate these phthalates from the excretory system as phase II conjugated compounds (glucuronide conjugate) [2] (Figure 3).

## 6. Pathogenesis of Liver

The liver is the largest organ and gland, a mass of two lobes in the human body that plays many vital roles for overall good health. The liver is a storage site for various macro-molecules, like protein and fat. It plays a crucial role in the detoxification of blood and synthesizing carbohydrates, blood coagulation factors, protein, triglyceride, and other biomolecules that are very important for the regulation of human physiology. One of the liver’s main functions is to secret bile salt, which is essential for the digestion of fat and fat-soluble bioorganic molecules in the small intestine. The excessive accumulation of toxins and fat within the liver leads to the emergence of metabolic diseases, such as non-alcoholic steatohepatitis (NASH), non-alcoholic fatty liver disease (NAFLD), steatosis, and non-communicable diseases [85,86]. The epidemiological and toxicological study of Migliarini et al. (2011) [87] has suggested that various phthalic acid esters induce numerous metabolic diseases, including enzyme metabolic disorders, atherosclerosis, NAFLD, and insulin resistance. These phthalates reduce the differentiation of adipose tissues and increase the number of lipid storage tissues, adipocytes, and induce lipidosis in the liver [88].

Lipids like triglycerides (TGs) are emulsified by bile acid and hydrolyzed by pancreatic lipase in the intestinal lumen to syn-2 monoacylglycerol and free fatty acids. The excessive glucose is converted into fatty acids via de-novo lipogenesis. When glycolysis increases, the hypersynthesis of citric acid from the citric acid cycle occurs due to which the fatty acid synthesis pathway becomes hyperactive, and the newly synthesized free fatty acids accumulate in the liver and induce metabolic disorders [89,90]. When glycolysis increases, the three-carbon-containing compound, phosphoenolpyruvate (PEP), is converted into pyruvate (C_3_). It generates one ATP with the help of a sex-dependent dimorphic enzyme, i.e., liver pyruvate kinase-4 (LPK-4). The LPK-4 is highly active in males as compared to females because of the presence of a high concentration of testosterone in males. LPK-4 is responsible for mitigating various metabolic disorders, such as lipidosis, lipogenesis, fatty acid synthesis, NAFLDs, and cholesterol biosynthesis, in males. However, females remain unaffected because of the low activity of LPK-4 in them. The three-carbon compound, pyruvate, increases because of overexpression of glycolysis. It is converted into the two-carbon compound, i.e., acetyl-CoA in the presence of the liver pyruvate dehydrogenase (PDH) complex, which is present in the inner mitochondrial membrane. It generates one molecule of NADH while converting pyruvic acid into acetyl-CoA by the PDH complex [91]. This acetyl-CoA acts as a substrate for Kreb’s cycle (anaplerotic cycle), which is responsible for the activation of fatty acid synthesis and its accumulation in the liver, which may cause metabolic disorders. Pyruvate dehydrogenase kinase-4 (PDK-4) is the regulatory enzyme for PDH, i.e., PDK-4 inhibits the activity of PDH. Breher-Esch et al. (2018) [92] have indicated that phthalate tends to inhibit the activity of PDK-4 and increase the activity of LPK-4 and de-novo-lipogenesis in the hepatocytes.

## 7. Effect of Phthalates on Hepatic Biomarkers of Oxidative Stress (OS)

The liver plays a significant role in detoxifying xenobiotics and toxins, including many other environmental pollutants. The liver’s detoxification process involves various enzymes of Phase I and Phase II biotransformation pathways. The imbalance between reactive oxygen species (ROS) and the body’s ability to detoxify these hazardous byproducts is known as oxidative stress (OS). Therefore, the monitoring of OS in the liver is significant because it can indicate how well the liver is functioning.

In mammals, a healthy liver contains certain marker enzymes such as alanine transaminase (ALT), aspartate transaminase (AST), lactate dehydrogenase (LDH), alkaline phosphatase (ALP), gamma-glutamyl transferase (GGT); enzymatic antioxidants such as superoxide dismutase (SOD), catalase (CAT), glutathione peroxidase (GPX), glutathione reductase (GR), glutathione-s-transferase (GST), lipid peroxidase (LPO); and non-enzymatic anti-oxidative molecules such as glutathione/reduced glutathione (GSH), vitamin C and vitamin E. Antioxidants are the molecules that delay or inhibit the oxidative process, which occurs due to excessive production of free radicals or reactive oxygen species (ROS) [93]. When the liver is exposed to xenobiotics like phthalates, the levels of these hepatic marker enzymes are altered, which induces OS [94]. Studies have shown that phthalates exhibit hepatotoxic potential as tested in various animal models such as mice [95], zebrafish [96], and quail [97].

Some findings also introduce new evidence suggesting that phthalates such as DEHP, mono- (carboxy isononyl) phthalate (MCNP), and mono-ethyl phthalate (MEP), may have a direct correlation with the levels of human ALP, ALT, GGT, and total bilirubin (TBIL) in terms of toxicity [98].

It has been reported that persistent OS due to the exposure of phthalates can cause an imbalance between the levels of endogenous and exogenous reactive oxygen species (ROS) leading to enhanced lipid peroxidation, reduction in antioxidant defense, and induced oxidative damage. Previous studies [95,96,97,98,99,100,101,102,103] have demonstrated that some phthalates like DEHP inhibit the activities of antioxidative enzymes and monooxygenase activity, causing a significant increase in the ROS level (Table 3). Phthalates (DEHP, MEHP, and butyl cyclohexyl phthalate (BCP)) are reported to adversely influence the levels of liver biomarkers (GSH, bilirubin), which may evoke the OS causing oxidative damage. The OS generated by BCP, DEHP, and MEHP may lead to histological and physiological changes in the hepatic tissues [104,105]. A study conducted by Yavasoglu et al. (2014) [105] administered an oral dosage of BCP (100, 200, and 400 mg/kg/day) to mice, and they observed toxic repercussions on the hepatocytes, along with a decline in the activities of antioxidant enzymes at a dose of 200 mg/kg/day. They further observed a rise in lipid peroxidation and hepatocyte degeneration, as well as the formation of vacuoles. The extent of lipid peroxidation was noted to be positively correlated with BCP concentrations [105]. The BCP can induce hepatocarcinoma with increasing concentrations of BCP, and BCP can induce lipid peroxidation, degeneration of hepatocytes, lipid droplet formation, and vacuole formation in the liver [105]. These conditions can generate hepatocarcinogenesis in the organism.

Previous research indicated that the liver was the primary target organ for DEHP and its metabolites, specifically mono 2-ethylhexyl phthalate (MEHP) [106]. The manifestation of hepatomegaly is considered the most prominent phenotype following exposure to DEHP; moreover, various adverse effects, such as apoptosis, hepatocellular carcinoma, necrosis, inflammation, and fibrosis, have been observed both in vitro and in vivo [104,105,106,107,108,109]. It has been demonstrated in many studies that the occurrence of OS is triggered by an overproduction of ROS. In many studies, it has been found that a DEHP-exposed liver is efficiently capable of inducing OS due to the triggering of the overproduction of ROS [95,110,111,112]. Phthalate (DEHP) exposure can modulate the inflammatory response and has been associated with the progression of non-alcoholic fibrosis and necrosis in the liver of rats [95].

Phthalates induce oxidative stress and cell degenerative processes by increasing the free radicals. Long-time exposure to phthalates suppresses the activities of the antioxidant enzymes and increases ROS, lipid peroxidase/thiobarbituric acid-reactive substance (TBARS), and malondialdehyde (MDA), leading to oxidative damage. The level of the antioxidant cytoprotective system decreases with long-term exposure to phthalate. In contrast, the levels of oxidative damage markers, including ALT, AST, MDA, LPO, and LPX, increase. Some other studies have clarified that the phthalates show their adverse effect on the levels of hepatic antioxidative enzymes of rat liver in a concentration-dependent manner, i.e., at a higher oral dose (200 or 400 mg/kg/day), phthalates significantly decrease the activities of CAT and SOD and the level of TBARS significantly increases [105].

Newsome et al. (2018) [113] found that when the liver is exposed to phthalates, the levels of hepatic ALT and AST increase, which may induce damage to the liver parenchymal cells. Both ALT and AST are released and circulated into the blood after the hepatocyte injury. The higher value of the ALT/AST ratio demonstrates damage to the liver. Zhao et al. (2019) [95] have elucidated the impact of DEHP on hepatic anti-oxidative enzymes and other markers in their study. They have demonstrated that, upon exposure to DEHP, the liver triggers the generation of reactive oxygen species (ROS) in excess, which may disrupt the levels and functions of superoxide dismutase (SOD), catalase (CAT), glutathione peroxidase (GPX), glutathione S-transferase (GST), and malondialdehyde (MDA).

The research conducted by Han et al. (2022) [114] reported that exposure to DEHP increased the ferroptosis in liver cells. Ferroptosis is a form of cell death that relies on iron and is characterized by the buildup of lipid peroxides. Emerging research points to its role in various liver diseases, such as non-alcoholic steatohepatitis (NASH), medicinal hepatic failure, and hepatic fibrosis. Oxidative stress is recognized as a primary driving factor for ferroptosis. Glutathione peroxidase 4 (GPx4) serves as a crucial regulator of ferroptosis, as it mitigates cellular oxidative stress by reducing phospholipid hydroperoxides in cell membranes. Both in vivo and in vitro investigations have revealed that inhibiting GPx4 can initiate ferroptosis in various cell types. Increased levels of intracellular iron foster the creation of ROS, which in turn induces oxidative stress and facilitates ferroptosis.

Some workers have discovered that the occurrence of particular phthalates in urine was positively and significantly associated with elevated levels of serum c-reactive protein (CRP) and gamma-glutamyl transferase (GGT) [81]. Both CRP and GGT serve as indicators of inflammation and oxidative stress, revealing gender-specific variations; for instance, females exhibit elevated levels of serum CRP and reduced levels of GGT levels compared to males. The levels of CRP and GGT were observed to rise with age [115,116]. The findings presented by these authors have indicated a significant correlation between occupational contact with phthalates and the occurrence of disturbances in the hepatic redox potential and its functions.

**Table 3:** List of phthalates associated with various diseases.

Name	Molecular Formula	Molecular Weight	CAS No.	Molecular Structure	Diseases/Parameters
Diisononyl phthalate (DiNP)	C_26_H_42_O_4_	418.61	28553-12-0	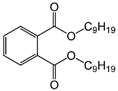	Induce oxidative stress and hepatic damage ^1^ [117]
Dicyclohexyl phthalate (DCHP)	C_20_H_26_O_4_	330.42	84-61-7	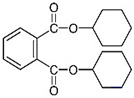	Immunotoxicity ^1^ [118], cardiovascular disease ^1^ (CVD) [119], reproductive toxicity ^1^ [120]
Diisopentyl phthalate (DIPP)	C_18_H_26_O_4_	306.40	605-50-5	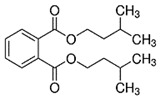	Disrupt the circadian rhythm ^1^ [121]
Diethyl phthalate (DEP)	C_12_H_14_O_4_	222.24	84-66-2	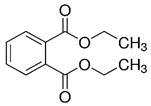	liver tumors ^1^ [122], hepatic and reproductive disorders ^1^ [123]
Dimethyl phthalate (DMP)	C_10_H_10_O_4_	194.18	131-11-3	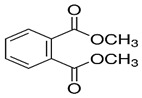	Anemia ^1^ [82], Hypercholesterolemia ^2^ [124]
Diallyl phthalate (DAP)	C_14_H_14_O_4_	246.26	131-17-9	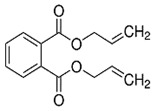	Necrosis ^1^, fibrosis ^1^ [125]
Butyl benzyl phthalate (BBP)	C_19_H_20_O_4_	312.36	85-68-7	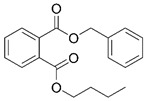	Oxidative damage ^1^, neurotoxicity ^1^ [126], developmental toxicity ^2^, oxidative stress ^2^ [127].
Di(2-ethylhexyl) phthalate (DEHP)	C_24_H_38_O_4_	390.56	117-81-7	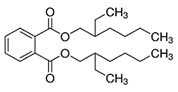	Antioxidative enzyme activity ↓, liver cell damage ^1,2^ [128], lipid accumulation ↑ ^1,2^, fatty acid synthesis↑ ^1,2^, NAFLDs ^1,2^ [129,130] Asthma [131], papillary thyroid cancer ^2^ [132], Thyroid Cancer ^2^ [133], liver tumor ^2^ [134], Leydig cell tumors ^1^ [135]
Diisodecyl phthalate (DIDP)	C_28_H_46_O_4_	446.66	26761-40-0	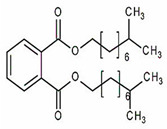	Testicular toxicity ^1^, male hypogonadism ^1^ [136]
Mono(2-ethylhexyl) phthalate (MEHP)	C_16_H_22_O_4_	278.34	4376-20-9	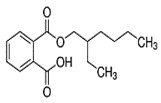	NAFLD ^2^, O.S. ↑ ^2^ [129] Thyroid Cancer ^2^ [133],
Diphenyl phthalate (DPP)	C_20_H_14_O_4_	318.32	84-62-8	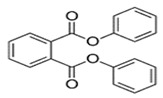	Inhibits cell proliferation ^2^ [137]
Diheptyl phthalate (DHP)	C_22_H_34_O_4_	362.50	3648-21-3	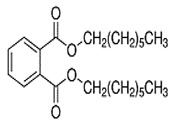	Disrupts the circadian rhythm, hepatic disorders, oxidative stress ^1^ [138]
Dibutyl phthalate (DBP)	C_16_H_22_O_4_	278.34	84-74-2	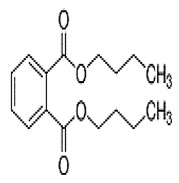	Liver metabolic disorder, hepatic toxicity ^1^ [139], Induce reproduction toxicity ^1^ [140], genotoxicity ^2^ [141] Hypertension^2^ [124]
Mono-methyl phthalate (MMP)	C_10_H_9_O_6_	180.16	4376-18-5	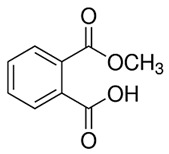	Increases fasting plasma glucose, insulin, high body, and trunk fat percentages, BMI, and body weight ^2^ [142], obesity ^2^ [143], lipogenesis ^1^ [144]
Benzyl butyl phthalate (BzBP)	C_19_H_20_O_4_	312.36	85-68-7	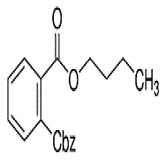	Rhinitis, eczema ^2^ [131], genotoxicity ^2^ [141] Disrupts the circadian rhythm ^1^ [121]
Mono-butyl phthalate (MBP)	C_12_H_14_O_4_	222.24	131-70-4	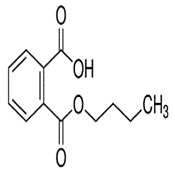	Thyroid Cancer ^2^ [145], disturbance in the redox balance of erythrocytes ^2^, genotoxicity ^2^ [141]
Di-n-octyl phthalate (DnOP)	C_24_H_38_O_4_	390.56	117-84-0	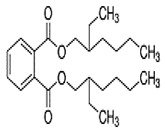	Reproductive toxicity, altered behavior, life span, lipid metabolism alteration, hypogonadotropic hypogonadism, inhibits spermatogenesis ^1^ [146]
Didecyl phthalate (DIDP)	C_28_H_46_O_4_	446.66	84-77-5	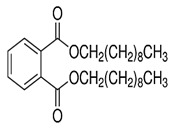	Oxidative stress, imbalance of cellular redox homeostasis ^2^ [147]
Mono-benzyl phthalate (MBzP)	C_15_H_12_O_4_	256.25	2528-16-7	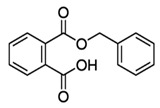	Thyroid Cancer ^2^ [145], genotoxicity ^2^ [124]
Diisobutyl phthalate (DiBP)	C_16_H_22_O_4_	278.34	84-69-5	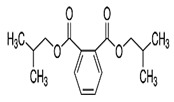	Disrupts the circadian rhythm ^1^ [121]
Monoisobutyl phthalate (MiBP)	C_12_H_14_O_4_	222.24	30833-53-5	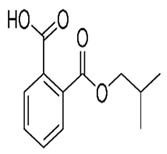	Cryptorchidism, fetal toxicity, testicular cancer, hypospadia ^1^ [148], cardiovascular disease ^2^, type 2 diabetes ^2^ [124]
Dipropyl phthalate (DiPP)	C_14_H_18_O_4_	250.29	131-16-8	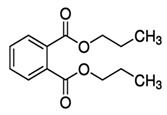	Hepatic, reproductive, and developmental toxicity ^2^ [149]

CAS No.: Chemical abstract number. Note: ^1^. Indicated about the animal model studies, ^2^. Indicated about the human studies.

## 8. Effect of Phthalates on Different Signaling Pathways

Zhang et al. (2021) [124] proposed that the induction of different lipid storage signaling pathways, including GPAT, SERBP, and FAS, by phthalates leads to the disruption of hepatic function. The higher concentrations of phthalates like DEHP could promote cellular apoptosis and lipid aggregation via the activation of PPARα and sterol regulatory element t-binding protein (SREBP-1c) signaling pathways, with the production of OS. The N-terminal of SREBP-1c enters into the nucleus and induces the biosynthesis of fatty acids, cholesterol, and lipids [150,151].

Zhang and colleagues (2021) [139] utilized SD rats as experimental subjects to investigate the detrimental impact of DBP on normal rat livers. Their study revealed that following exposure to DBP, there was a notable elevation in the activities of SREBP1 and FAS proteins in rat livers, with a significant reduction in the expression of p-AMPK and PPAR-α proteins. Typically, SREBP-1c and its associated fatty acid synthesis pathway exhibit minimal expression and functionality in healthy tissues but become activated in tissues exhibiting abnormal lipid metabolism due to DBP exposure. Additionally, a decline in the activity of antioxidative enzymes was also observed, with a rise in the levels of triglyceride (TG), total cholesterol (TC), ALT, AST, and ALP. These outcomes suggest that DBP potentially promotes lipid accumulation and disrupts the metabolic equilibrium of NAFLD by activating the SREBP1-c/FAS signaling pathways. Essentially, acting as a PPARα agonist, DBP may exacerbate the advancement of NAFLD. This assertion aligns with prior research indicating that DBP directly interacts with PPARα, with the activation of the PPAR receptor representing a pivotal mechanism through which DBP induces hepatotoxic effects [152].

Furthermore, several investigations have been conducted to assess the influence of phthalates on PPAR receptors and their associated signaling pathways [120]. The majority of hepatic genes altered by DEHP have been found to be targets of PPARα. Following exposure to DEHP and its metabolite, phthalic acid, in primary hepatocytes of zebrafish, all PPARs exhibited an increase, albeit dependent on sex and dosage. These authors have highlighted the significance of PPARs in the rapid assessment of DEHP and the presence of phthalic acid. Some workers have observed that PPARα could be activated by DEHP and BBP, as evidenced by the upregulation of PPARα target genes, such as cytochrome P (CYP) 4a12b, CYP4a14, and Acot1 [153].

In signaling, phosphorylation is the most important phenomenon that stimulates or activates different biomolecules involved in signaling pathways. According to some researchers [139,154,155,156], phosphorylation of adenosine monophosphate-activated protein kinase (AMPK) is responsible for the inhibition of fatty acid synthesis. The phosphorylated AMPK (pAMPK) induces β-oxidation of fatty acid into the mitochondria of adipose tissues and liver [157,158]. Phthalates (DBP) dephosphorylate pAMPK, which promotes obesity, hyperlipidemia, cholesterol storage, TG synthesis, and the deposition of free fatty acids in adipocytes and hepatocytes, known as NAFLD. These phthalates interact with the GPCR of the plasma membrane and directly stimulate the activity of PPAR. There are three types of PPAR in nature: PPAR-α, PPAR-β, and PPAR-γ. Among all these three signaling molecules, PPAR-α is the most active and controlling element as compared to others. The PPAR-α, PPAR-β/γ, FAS (fatty acid synthase), GPAT, SREBP-1c, and AMPK signaling influence the upregulation of lipid, triglyceride, and fatty acid synthesis in the liver and play a significant role in the pathogenesis of NAFLD [152,159]. Thus, phthalates hyperactivate the lipid metabolism-related genes, which promote the differentiation and maturation of adipocytes, causing the development of a fatty liver [160,161,162].

In recent years, research has demonstrated that the activated JAK2/STAT5 within the hepatic environment positively regulates the expression of peroxisome proliferator-actiated receptor gamma (PPARγ). The significance of PPARγ as a pivotal contributor to lipogenesis is widely acknowledged. The impact of JAK2/STAT5 on hepatic lipid metabolism appears to be mediated through the regulation of PPARγ expression. Moreover, the JAK2/STAT5 pathway exhibits the capacity to directly modulate the expression of crucial hepatic lipid metabolism-associated elements like FAS and HSL. STAT5 can directly affect the FAS levels. Researchers have demonstrated that FAS promotes the production of long-chain fatty acids, which is associated with fatty acid metabolism [163,164]. These discoveries underscore the pivotal involvement of the JAK2/STAT5 signaling pathway in hepatic lipid metabolism.

**Figure 4:**
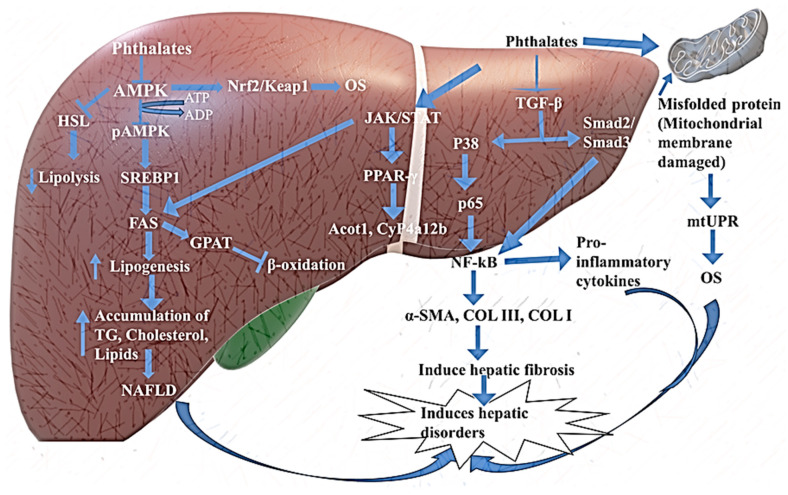
A schematic illustration delineating the toxicological impact of phthalates in the liver and its molecular mechanisms on the onset of inflammation and oxidative stress. This figure suggested that phthalates alleviated oxidative stress and inflammatory pathways and modulated the signaling cascades associated with DNA damage. AMPK: adenosine monophosphate-activated protein kinase, ATP: adenosine tri-phosphate, pAMPK: phosphorylated adenosine monophosphate-activated protein kinase, SREBP: sterol regulatory element-binding protein, FAS: fatty acid synthase, HSL: hormone-sensitive lipase, GPAT: glycerol-3-phosphate acyltransferase, TG: triglyceride, NAFLD: non-alcohol fatty liver disease, TGF-β: transforming growth factor-beta, JAK/STAT: janus kinase-signal transducer and activator of transcription. PPAR-γ: peroxisome proliferator-activated receptor gamma, NF-kB: nuclear factor kappa B, α-SMA: alpha-smooth muscle actin, COL III and I: collagen type III and I, Acot1: acyl-CoA thioesterase 1.

Some studies have shown that long exposure to phthalates may induce the overproduction of ROS, which can impair many signaling pathways such as JAK2/STATE5 pathways, and play a key role in the regulation of lipid accumulation, adipogenesis, and lipogenesis in the liver [165] when an organism is exposed to these phthalates (MEHP). They inhibit JAK2/STATE5 signaling, causing lipogenesis and inducing FA-synthesis in the liver and other adipose tissue. Some studies have shown that chronic exposure to phthalates might cause an increase in reactive oxygen species (ROS), leading to the potential impairment of various signaling cascades such as the JAK2/STATE5 pathways. These pathways are crucial in regulating processes like lipid accumulation, adipogenesis, and lipogenesis within the liver. When an organism comes into contact with these phthalates (MEHP), they disrupt JAK2/STATE5 signaling by the disruption of JAK2 in adipose, resulting in increased lipogenesis and fatty acid synthesis in the liver, TG, and other adipose tissues. Phthalates disrupt the JAK2 in adipose tissue and could increase the TG. Lipogenesis causes the liver to become fatty. This may be due to the downregulation of lipolysis and by decreasing the activity of the mitochondrial function Figure 4 [136,166].

Reactive oxygen species (ROS) induce hepatic cellular injury, leading to the secretion of inflammatory mediators, which subsequently recruit immune cells to enhance the inflammatory response by generating pro-inflammatory cytokines and growth factors. Oxidative stress assumes a pivotal role in initiating these inflammatory and fibrotic reactions. Transforming growth factor-β1 (TGF-β1) serves as a significant cytokine in the fibrogenesis process, playing a crucial function in converting hepatic stellate cells (HSCs) into fibroblasts and stimulating extracellular matrix (ECM) synthesis. TGF-β1 primarily triggers the phosphorylation of Smad2 and Smad3 via binding to the TGF-β type I receptor, consequently promoting fibrosis. The suppression of TGF-β1 activity, affecting both Smad2 and Smad3, significantly alleviates hepatic fibrosis in numerous animal models of fibrosis. Zhao and colleagues found that DEHP exposure promoted the up-regulation of TGF-β1, P-Smad2, and P-Smad3 following liver damage. DEHP exposure can stimulate the expression of inflammatory proteins COL-I, COL-III, α-SMA, P-p38, and P-p65 by TGF-β1 and NF-kB pathways [95].

Researchers found that, when the organism is exposed to phthalate (DEHP), lipids accumulate in the hepatic tissue, and these cells inflammatory factors IL-6, IL-1β, and TNF-α increase in serum, and the adipose tissue of an organism induces inflammation in the organism [88]. The inflammatory effects of phthalates may be mediated through actions of the nuclear factor kappa-light-chain-enhancer of activated B cells (NF-kBs). Phthalate exposure may activate NF-kB, which signals the production of pro-inflammatory cytokines, such as interleukin-6 (IL-6), and TNF-α [167,168,169,170]. NF-kB also stimulates the production of c-reactive protein (CRP), a marker of systemic inflammation mediated via IL-6 and IL-1β [171]. Additionally, phthalates may trigger inflammatory responses through their interactions with the estrogen receptor [172] or PPARs [104,173], as well as the induction of oxidative stress [174].

Numerous studies have demonstrated a significant correlation between the generation of ROS and mitochondria, playing a pivotal role in the regulation of apoptosis. As a result, exposure to high levels of monobutyl phthalate (MBP) can lead to an excess of ROS production in the hepatic oxidative system, causing cytotoxic effects and a decrease in cellular viability. In a study by Jiao et al. (2020) [96], the deleterious impact of MBP was evaluated in the liver of Zebrafish using varying concentrations (0, 0.5, 5, and 10 mg/L) at different time intervals, up to 96 h. They have observed significant alterations in apoptotic markers at 10 mg/L through the suppression of antioxidative genes (HO-1SOD, CAT, Nrf2, and GPx), along with a concomitant upregulation in the expression of apoptotic genes (p53, Cas3, and Bax).

Phthalates, such as DEHP and DBP, are widely found in many parts of the ecosystem, including contaminated marine life [175], rivers [176], plants [177], air, and soil [178]. Prior studies have demonstrated that exposure of experimental rats to DEHP resulted in a decrease in the weight of the liver [179] and triggered hepatotoxicity by upregulating the activities of acidic and alkaline ceramidases [180]. A previous study demonstrated that DEHP promoted the synthesis of phospholipids that were potentially associated with the proliferation of peroxisomes within the hepatic tissue [181].

Previously it has been proven that phthalates can decrease the activity of liver antioxidative enzymes by the alteration in the catalytic active site of enzymes. These phthalates potentially increase ROS production and reduce the expression of antioxidative enzymes (SOD and GPx) [182]. The phthalates have the potency to disturb the homeostasis of organisms by disturbing the redox potential at the cellular level. Excess ROS accumulation leads to excessive lipid peroxidation and free radical damage in the body.

The Nrf2/Keap1 pathway controls various genes and functions involved in oxidative stress management, inflammation, DNA damage recognition, and many other processes [183]. Normally, oxidative stress stimulates and activates the Nrf2/Keap1 pathway in a dose-dependent manner. Nrf2 plays a significant role in preserving the fundamental mechanism of the hepatic antioxidant defense system. Inhibition of Nrf2 increases lipid peroxidation [184] and regulates both basal and induced expression of detoxifying antioxidant enzymes [104]. The overexpression of Nrf2 has demonstrated a protective function in relieving oxidative stress. It has been observed that DEHP has the potential to enhance Nrf2-mediated oxidative stress in quail liver in a dose-dependent manner. The prolonged accumulation of Nrf2 had a detrimental impact, exacerbating oxidative stress [185].

The liver is the main target of action for exogenous toxicants/xenobiotics. Wang et al., (2015) [186] and Sicińska et al. (2021) [141] have identified that exposure to DEHP, di-n-butyl phthalate (DBP), butylbenzyl phthalate (BBP), mono-n-butyl phthalate (MBP), and mono-benzyl phthalate (MBzP) shows genotoxicity in the organism. Exogenous toxicants primarily target the mitochondria. These toxicants cause the misfolding of proteins and the entry of these misfolded proteins into mitochondria, severely increasing stress and causing damage [187]. The OS causes mitochondrial malfunction and triggers a protective unfolded protein response (mtUPR) in RPE cells [187,188]. The misfolded proteins accumulate in the intermembrane of mitochondria and prompt the mitochondrial membrane space by increasing the expression of certain genes such as high-temperature requirement protein A2 (HtrA2) [189]. Exposure to high concentrations of DEHP blocks mtUPR and exacerbates mitochondrial damage. The mtUPR is the main factor in repairing the damage or reducing OS introduced by mitochondrial damage. Some authors have suggested that a high concentration of DEHP induces hepatotoxicity by the dysfunction of the Nrf2 signaling pathway and mtUPR elements. However, further studies are required to evaluate the adverse impact of phthalates on different signaling pathways, leading to hepatic metabolic disorders.

## 9. Therapeutic Strategies for Phthalate-Mediated Alteration in the Hepatic System

The management of the toxicity of phthalates in the biological systems may be conducted through (1) prevention from exposure to phthalates, (2) removal of phthalates from the body, and (3) use of dietary supplements, including plant-based bioactive compounds [190].

The prevention strategy may include avoiding the use of plastic containers in microwaves, vinyl toys, perfumed shampoo, and lotion [52]. The use of fragrance-free products should be encouraged. Instead of processed canned foods, fresh foods should be preferred to avoid exposure to phthalates. The process of the removal of phthalates from the body involves a combination of different ways, such as sweat, urine, feces, liver, and gastrointestinal tract [191]. Preventing exposure to phthalates is the most effective and protective strategy, rather than dietary supplements. This strategy includes reducing the use of phthalate-containing products, increasing public awareness about phthalate sources in daily life, and improving environmental regulation. It is known that phthalates are pervasive in the environment, and reducing exposure would not only protect against liver injury but also decrease the risk of other systematic toxicities [192,193].

In addition, some plant-based bioactive molecules have been reported to help mitigate phthalate-mediated toxicity. Some of these phytochemicals are quercetin, lycopene, ellagic acid, genistein, selenium compounds, and many others [194]. Quercetin, a flavonoid found in various fruits and vegetables, possesses anti-inflammatory, antioxidant, and estrogenic properties. Studies have demonstrated that quercetin administration in rodent models exposed to phthalates diminished the hepatic oxidative stress and preserved liver histology [195]. It can reverse the phthalate-mediated male reproductive toxicity in rats. Apigenin, another flavonoid, was found to reduce DEHP-induced ROS-mediated ferroptosis, enabling liver cells to maintain their lipid peroxidation and iron homeostasis [114]. Ellagic acid is an antioxidant and can help reduce the OS and inflammatory reactions [196]. Genistein acts as an antioxidant and can mitigate phthalate-mediated reproductive toxicity by increasing the Nrf2 pathways and restoring hormonal balance [197,198]. Curcumin, a polyphenol from *Curcuma longa*, is known for its antioxidant and anti-inflammatory properties. Curcumin has been shown to counteract oxidative stress by scavenging ROS and upregulating endogenous antioxidant defense systems, such as CAT, and SOD. Tsai et al. (2015) [199] demonstrated that curcumin is used as a therapeutic agent for preventing DEHP-associated liver cancer progression. Curcumin effectively deduced the DEHP-mediated hepatotoxicity by mitigating the cell migration, invasion, and epithelial-mesenchymal transition (EMT). It decreased the population of cancer stem cell (CSC)-like cells in hepatocellular carcinoma (HCC) cell lines in vitro and impedes tumor growth and metastasis in vivo. Silymarin and rutin hydrate offer protective benefits against liver injury through antioxidant, anti-inflammatory, hepatoprotective, immunomodulatory, anti-tumor, and cell-regulating pathways [200,201,202]. It has been demonstrated that silymarine and rutin attenuate phthalate-induced liver injury by enhancing mitochondrial function and decreasing the level of oxidative stress. Their hepatoprotective effects have been attributed to their ability to modulate NF-κB and MAPK signaling cascades. Their efficacy against DEHP toxicity and hepatic encephalopathy makes them valuable therapeutic agents for liver health management [203,204,205,206]. Apium, spirulina, resveratrol, and ciliary oil are known for their antioxidant and anti-inflammatory effects. They significantly inhibit the initiation, promotion, and tumor progression and protect from genotoxicity. Consequently, these phytochemicals are utilized in the treatment of oxidative damages induced by DEHP [207,208,209].

Some flavonoids and polyphenols extracted from Mangifera oleifera have been reported to possess anti-inflammatory, hepatoprotective, antioxidant, and neuroprotective characteristics [210,211,212]. The application of this extract may decrease oxidative stress-related levels of NF-κB and apoptosis [213,214]. Recent findings suggest that the extracts from this plant may activate phase II detoxification mechanisms, particularly the Nrf2/HO-1 antioxidant pathway. The activation of Nrf2 by MO leads to the activation of detoxifying enzymes, including various isoforms of glutathione S-transferase (GST) P, Pi, and A1, heme oxygenase-1 (HO-1), and NAD(P)H: quinone oxidoreductase 1 (NQO1) [215]. Amara et al. (2021) [216] noted that MO mitigates DEHP-induced endoplasmic reticulum (ER) stress in SH-SY5Y cells by preserving the balance of ROS and aiding the function of mitochondrial respiratory chain complexes [111].

Lycopene, a carotenoid compound, is a potential antioxidant, which can ameliorate phthalate-induced oxidative damage and preserve mitochondrial function [217]. The study by Gad et al. (2023) [218] indicated that carotenoids like lutein can decrease DEHP-induced hepatic injury in rats by reducing the elevated levels of biomarkers associated with liver injury, including cystatin-C, α-fetoprotein, and inflammatory markers. The Selenium compounds also have antioxidant properties and can protect the phthalate-exposed organism from oxidative damage by improving the activities of antioxidant enzymes [219]. The present study is summarized in graphical abstract.

## 10. Conclusions

The prolonged exposure to phthalates can adversely impact almost all the organs of the exposed animals and humans. The liver, being a primary organ responsible for the detoxification and biotransformation of xenobiotics, is largely affected by the phthalates and their metabolites leading to hepatic congestion, oxidative stress (OS), inflammation, and metabolic disruptions. Phthalates interfere with lipid metabolism and energy homeostasis, mediated by nuclear receptors such as PPARs and AMPK, resulting in perturbations in fatty acid metabolism and inflammation signaling cascades, notably via activation of NF-kB, which exacerbates oxidative stress and immune dysregulation. Phthalates lipophilicity, reflected in their logP values, correlated with their bioaccumulation potential and interactions with cell membranes and metabolic enzymes. Higher logP values are often associated with prolonged retention in lipid-rich tissue, amplifying their toxicological impact. The phthalates-mediated generation of ROS further compounds the hepatic damage and promotes lipid peroxidation, mitochondrial dysfunction, and impaired detoxification. There is an urgent need to conduct extensive research on the toxicological effects of phthalates and their metabolites to investigate the relationship between physiochemical properties and bioaccumulation. Detailed studies on the modulation of nuclear receptors are essential to understanding the molecular bases of phthalate-induced endocrine disruption. Moreover, extensive demographic and epidemiological studies are necessary to capture exposure patterns and associated health risks across diverse populations. Though the role of some plant-based bioactive molecules has been reported to ameliorate phthalates-mediated toxicity in experimental animals primarily via a decrease in ROS and oxidative stress, the phytochemicals with high efficiency and least toxicity are yet to be discovered.

## Figures and Tables

**Table 1:** Some physical properties of phthalates at 25 °C.

Phthalates	Partition Coefficient		Melting Point (°C)	Boiling Points (°C)	Physical State of Phthalate	Appearance (Colour)
	LogP	K_ow_				
Diisononyl phthalate	9.6	9.3	−48	252 °C	liquid	colorless
Dicyclohexyl phthalate	5.6	6.2	66	224	solid	white
Diisopentyl phthalate	5.6	5.2	NA	NA	solid	colorless to dark Green-yellow
Diethyl phthalate	2.5	2.47	−40.5	295	liquid	colorless
Dimethyl phthalate	1.6	1.6	5.5	283.7	liquid	colorless
Diallyl phthalate	3.3	3.23	−70	290	liquid	light yellow
Butyl benzyl phthalate	4.9	4.73	−35	370	liquid	colorless
Di(2-ethylhexyl) phthalate	7.4	7.6	−55	384	solid	colorless
Diisodecyl phthalate	10.6	NA	−50	253	liquid	colorless
Mono(2-ethylhexyl) phthalate	4	NA	>94	>300	solid	white
Diphenyl phthalate	4.5	NA	74	NA	solid	white
Diheptyl phthalate	8	NA	NA	360	liquid	colorless
Dibutyl phthalate	4.7	4.5	−35	340	liquid	colorless
Mono-methyl phthalate	1.1	NA	82	NA	solid	beige
Benzyl butyl phthalate	4.9	4.73	−35	370	liquid	colorless
Mono-butyl phthalate	3.1	NA	73.5	NA	solid	white
Di-n-octyl phthalate	9.1	8.10	25	220	liquid	colorless
Didecyl phthalate	7.5	9.05	2.5	233	liquid	light yellow
Mono-benzyl phthalate	3.3	NA	NA	NA	solid	white
Diisobutyl phthalate	4.1	4.11	−64	296	liquid	colorless
Monoisobutyl phthalate	2.3	NA	108	NA	solid	white
Dipropyl phthalate	4	NA	−31	317	liquid	light yellow

Compounds with an optimal LogP range are more likely to penetrate cell membranes effectively, which can accelerate their biological activity. K_ow_ is supposed to show phthalates’ environmental behavior and potential health risks. It is used to find the potential of phthalates to cause toxic effects within cells. Not available.

**Table 2:** Summary of detected phthalates/metabolites, and their concentration ranges in human biological samples.

Parent Phthalates	Phthalate Metabolites	Biological Matrices	Concentration Range (µg/L)	Primary Sources of Exposure
DEHP	MEHP, Mono (2-ethyl-5-oxohexyl) phthalate (MEOHP) Mono(2-ethylhexyl) phthalate (MEHP), Mono(2-ethyl-5-hydroxyhexyl) phthalate (MEHHP), Mono(5-carboxy-2-ethylpentyl) phthalate (MECPP) and Mono(2-carboxymethyl) hexyl) phthalate (MCMHP)	Urine, serum, Cord Serum, breast milk, Placental Tissue, Adipose Tissue, Sweat, Semen, saliva	Urine: 0.3–1300 µg/L [58] Serum: 0.1–10 µg/L [59]; Cord Serum: 0.1–8.4 µg/L [59,60,61] Breast milk: 0.49–13 µg/L [60,61]; Placental Tissue: 0.1–5 µg/kg; Adipose Tissue: 0.05–5 µg/kg [62]; Sweat: 0.2–20 µg/L [63]; Semen: 0.1 µg/L [64]; Saliva: 6.8 ng/mL [59]	Building products, food packaging materials, medical devices, raincoats, exposure from PVC products, food containers, car products, grip bumpers, wallpaper
Diisononyl phthalate (DiNP)	Monoisononyl phthalate (MiNP), Mono (carboxy isononyl) phthalate (MCiNP)	Urine, blood, breast milk	Urine: 0.2–50 µg/L Breast Milk: 101 µg/L [57]	Traps, PVC products, flooring materials, exposure from plastic materials, footwear, paper production, varnish and paint, pool liners, toys
Butylbenzylphthalate (BBzP)	Monobenzyl phthalate (MBzP)	Urine, saliva, blood, hair	Urine: 0.5–250 µg/L; Saliva: 353.6 ng/mL [65]; Blood: 0.82–1.97 ng/mL [66] Hair: 4 μg/mL [67]	Food conveyor, vinyl flooring, adhesives, synthetic leather, perfume, traffic cones
Diisodecyl phthalate (DiDP)	Mono(carboxyisooctyl) phthalate (MCiOP), Mono-isononyl phthalate (MiNP)	Urine	Urine: 0.3–3.3 µg/L [67]	some medical devices, cables, paints, adhesives, construction sites, plastic carpets, jackets, pool liners
Dihexyl phthalate (DHP)	di-*n*-hexyl phthalate	Urine	0.1–1.3 μg/L [68]	Plasticizer synthesis, PVC, cosmetics, plasticizers in consumer products
Diisobutyl phthalate (DiBP)	Mono-isobutyl phthalate (MiBP)	Urine	Urine: 1.5–103.9 µg/L [57,69]	Printing ink, paints, and adhesives used for the synthesis of plastics, carpet tiles
Dibutyl phthalate (DBP)	Mono-n-butyl phthalate (MBP), Mono-isobutyl phthalate (MiBP)	Urine, serum, Cord Serum, Sweat, umbilical cord blood, Adipose Tissue, Breast milk, Semen, saliva, peripheral blood, hair	Urine: 1.2–2540 µg/L [70]; serum: 0.1–3 µg/L [60]; Sweat: 0.1–10 µg/L [71] Umbilical cord blood: 0.019–5.71 µg/mL [72] Adipose Tissue: 0.05–3 µg/kg [63] Breast milk: 0.10–12 µg/L [57,61] Semen: 0.1 µg/L [64] Saliva: 17.9–65.8 ng/mL [65]; peripheral blood: 0.051–7.67 µg/mL [73]; Hair: 2 µg/mL [74]	Nitrocellulose lacquer, personal care products, adhesives, coatings, plastics, polymer synthesis, print ink, beauty care products, solvent for dyes
DEP	MEP	Urine, Serum, Cord, Serum, Sweat, Adipose Tissue, Breast Milk, Semen, Saliva	Urine: 4.2–1388 µg/L [57,68,70]; Serum: 0.1–5 µg/L [59,60] Cord Serum: 0.1–3 µg/L [59], Sweat: 0.1–10 µg/L [63], Adipose Tissue: 0.05–3 µg/kg [62]; Breast Milk: 0.05–1 µg/L [60]; Semen: 0.1 µg/L [64], Saliva: 91.4 ng/mL [65]	Personal care products (e.g., perfumes, lotions), cosmetics, industrial products such as herbicides, plasticizers, medicine coating, aerosol sprays
Dimethyl phthalate (DMP)	Monomethyl phthalate (MMP)	Urine, blood, saliva, serum	Urine: 1.2–33 µg/L [60,75] Serum: 0.5 µg/L, 3.4 ng/mL [60,76] Breast Milk: 0.1 µg/L [64] Blood: 2 ng/mL [76]: 1000 mg/kg [77] Saliva: 3.1 ng/mL [65]	Safety glass, cosmetic products (e.g., perfumes, hair sprays), Insect repellents

Note: Concentration ranges are approximate and may vary depending on population demographics, sources of exposure, and the analytical methods employed in the respective studies.

## Data Availability

Data sharing does not apply to this article. All data are contained within this manuscript.
